# Determining how best to support overweight adults to adhere to lifestyle change: protocol for the SWIFT study

**DOI:** 10.1186/s12889-015-2205-4

**Published:** 2015-09-04

**Authors:** Rachael W. Taylor, Melyssa Roy, Michelle R. Jospe, Hamish R. Osborne, Kim J Meredith-Jones, Sheila M. Williams, Rachel C. Brown

**Affiliations:** Department of Medicine, University of Otago, PO Box 56, Dunedin, 9054 New Zealand; Department of Human Nutrition, University of Otago, PO Box 56, Dunedin, 9054 New Zealand; Department of Preventive and Social Medicine, University of Otago, PO Box 56, Dunedin, 9054 New Zealand

**Keywords:** Obesity, Adherence, Mobile applications, Hunger, Self-weighing, Intermittent fasting, High-intensity interval training, Self-monitoring

## Abstract

**Background:**

Physical activity plays a critical role in health, including for effective weight maintenance, but adherence to guidelines is often poor. Similarly, although debate continues over whether a “best” diet exists for weight control, meta-analyses suggest little difference in outcomes between diets differing markedly in macronutrient composition, particularly over the longer-term. Thus a more important question is how best to encourage adherence to appropriate lifestyle change. While brief support is effective, it has on-going cost implications. While self-monitoring (weight, diet, physical activity) is a cornerstone of effective weight management, little formal evaluation of the role that self-monitoring technology can play in enhancing adherence to change has occurred to date. People who eat in response to hunger have improved weight control, yet how best to train individuals to recognise when true physical hunger occurs and to limit consumption to those times, requires further study.

**Methods/design:**

SWIFT (Support strategies for Whole-food diets, Intermittent Fasting, and Training) is a two-year randomised controlled trial in 250 overweight (body mass index of 27 or greater) adults that will examine different ways of supporting people to make appropriate changes to diet and exercise habits for long-term weight control. Participants will be randomised to one of five intervention groups: control, brief support (monthly weigh-ins and meeting), app (use of MyFitnessPal with limited support), daily self-weighing (with brief monthly feedback), or hunger training (four-week programme which trains individuals to only eat when physically hungry) for 24 months. Outcome assessments include weight, waist circumference, body composition (dual-energy x-ray absorptiometry), inflammatory markers, blood lipids, adiponectin and ghrelin, blood pressure, diet (3-day diet records), physical activity (accelerometry) and aerobic fitness, and eating behaviour. SWIFT is powered to detect clinically important differences of 4 kg in body weight and 5 cm in waist circumference. Our pragmatic trial also allows participants to choose one of several dietary (Mediterranean, modified Paleo, intermittent fasting) and exercise (current recommendations, high-intensity interval training) approaches before being randomised to a support strategy.

**Discussion:**

SWIFT will compare four different ways of supporting overweight adults to lose weight while following a diet and exercise plan of their choice, an aspect we believe will enhance adherence and thus success with weight management.

**Trial registration:**

Australian and New Zealand Clinical Trials Registry ACTRN12615000010594. Registered 8^th^ January 2015.

## Background

Overweight and obesity affect almost two-thirds of New Zealand adults and rates continue to climb in some ethnic and demographic groups [1]. The inability of many individuals to lose significant weight by themselves or for most to keep it off is due to a myriad of factors including making changes to lifestyle that are too drastic and therefore not sustainable, the widespread availability of energy-dense foods and sedentary past-times, changes to work and leisure-time energy expenditure, and insufficient support structures [2, 3]. However, considerable debate and confusion also exists in both lay and scientific literature regarding whether there is an “optimal” dietary composition for weight loss [4]. Low-carbohydrate diets in particular have long been purported to have metabolic and clinical advantages over other dietary patterns [5–7]. It makes mechanistic sense that lower carbohydrate diets *could* promote greater weight loss, principally because of a reduction in insulin levels reducing the storage of body fat [8]. However, at the practical level, recent meta-analyses suggest little difference in weight and health outcomes between diets differing quite markedly in macronutrient composition, particularly over time frames longer than 6 months [9–13]. Instead, a far more relevant factor appears to be the degree of *adherence* to the prescribed diet [11, 14, 15], an aspect that is insufficiently measured [16].

Such findings have led expert groups to recommend that the most suitable diets are not those that include a specific nutrient composition per se, but rather ones that entail moderate energy restriction which participants are willing and able to follow long-term [17]. It is becoming clear that several acceptable dietary patterns that differ quite markedly in terms of macronutrient composition are suitable for weight loss. The more important question then becomes how best to encourage and support long-term compliance with one, or more, of these patterns [18]. However, while adherence to dietary change is viewed as a cornerstone of non-communicable disease prevention and management [19], little practical guidance is available identifying which specific factors enhance adherence to dietary advice [3]. The short-term nature of most studies, marked differences in terms of how adherence is assessed, and overall low trial quality limits firm conclusions being drawn about the most efficacious factors [3].

Strong social support is considered an important adjunct to successful weight loss or maintenance [20]. The use of personalised support with nutrition and activity specialists is known to be effective [21], however it is expensive and rarely accessed. Alternative forms of low-intensity but regular support, delivered by non-specialists can result in similar benefits, at a fraction of the cost [22]. Brief monthly support, such as is found in many commercial weight loss programmes, also appears to maintain weight loss better over 2–3 years than other forms of support including interactive websites and self-directed control [23]. However, because such support cannot continue indefinitely, and ongoing development of innovative technology, determining the efficacy of other, low-cost strategies is currently of great interest [24].

Self-monitoring of weight, food intake and/or activity levels has been shown to be one of the most effective strategies employed for successful weight management [25]. However, monitoring of food and activity is time-consuming and adherence dramatically declines over time [26]. The advent of mobile “apps” may offer a more effective way of monitoring food/ activity patterns due to instant feedback of a wealth of information [27]. While self-monitoring strategies are a common component of the myriad of commercially available apps [24], whether they are effective at encouraging behaviour change has rarely been examined, despite their widespread use [28]. Although the top five self-monitoring apps each have a user base of more than 10 million people [28], only one appears to have been tested in a clinical trial [29]. Laing et al. [29] recently demonstrated that use of MyFitnessPal alone, with no accompanying dietary or exercise advice, did not produce significant weight change over 6 months in comparison with usual care. The apparent lack of effect may be due in part to the sharp declines in adherence to app use after the first month [29]. Perhaps this is not surprising given that the response of individuals to self-monitoring can vary considerably, ranging from the “well-disciplined” who endorse this approach, to those that have “diminished support”, where other co-existing factors take precedence [30].

Compared with monitoring of diet or activity, monitoring of body weight is both straightforward and quick. Although weekly or monthly weighing has traditionally been recommended, observational studies suggest that daily weighing may promote weight loss better than less frequent weighing [31–33]. However, few studies have examined the efficacy of daily self-weighing in comparison to less frequent weighing via randomised controlled trials. Steinberg et al. [34] reported a weight loss of 6.6 % over a 6-month period in the daily weighing group compared with only 0.4 % in wait-list controls. It may be that more frequent weighing offers further advantages; Oshima et al. [35] demonstrated that twice-daily weighing resulted in greater weight loss than once-daily weighing in a small group of overweight adults. How these changes related to changes in actual body composition is unknown, but important given that it is widely acknowledged that fat loss, rather than weight loss, provides the key health benefits [36]. Whether such frequent weighing produces adverse psychosocial effects is a matter of concern. However, the limited data to date suggest that no adverse effects, and potentially even some benefits, have been observed, at least over 6–18 months [37–39].

One of the major barriers to effective weight management is that we eat for a variety of complex and interrelated reasons other than hunger, including taste, social interaction and emotional cues. Observational studies demonstrate that many environmental and situational cues influence our eating [40], but those who eat in response to hunger, recognise satiety signals, and give themselves unconditional permission to eat foods of their choosing (intuitive eaters), are more likely to be a healthy weight than those who do not [41, 42]. While people can be trained to eat more intuitively (in response to hunger and satiety), whether this increases weight loss relative to other techniques remains uncertain [43, 44]. An alternative type of *hunger training* has been suggested, where subjects are trained over a few weeks to identify actual (physiological) hunger by connecting the physical feelings of hunger with blood glucose levels [45]. This training has been shown in one small study to produce significant weight loss compared with a conventional approach which required constant dietary restraint [46]. Whether this approach is a viable way of training individuals to eat to their appetites requires further examination, particularly in terms of its ability to work over longer time frames.

Testing the effectiveness of different support strategies should theoretically occur with all participants following the same diet. However, we know that one of the major difficulties in trials which randomise people to follow specific dietary patterns is that any particular diet cannot possibly suit every individual, which undoubtedly influences adherence. Behavioural choice theory posits that outcomes are improved when participants receive the treatment they prefer [47]. It thus seems feasible that tailoring a diet on the basis of individual personal and cultural preferences (i.e. choice) may therefore have the best chance for long-term success [48], perhaps through enthusiasm, a better fit with their overall lifestyle, or supporting personal autonomy. However, whether choice of intervention group versus randomisation affects adherence and outcomes in weight loss studies has been examined infrequently, and the findings do not generally support the theory [49]. There was no evidence of a difference in outcomes from having a preference for a certain intervention in terms of group versus individual treatment [50], low fat versus low carbohydrate diets [51], or choice of commercial diet programme [52]. The remaining study found significantly greater weight loss in those randomised to a diet than in those who were allowed to choose a diet, although clinically important weight losses were observed in both groups [53]. However, many of these studies were relatively small and were unable to provide precise estimates of effects [50, 52, 53], or lasted less than one year [50]. Thus further examination of the impact of being able to *choose* which diet or activity plan to follow is warranted, particularly given the high drop-out rates typically observed in randomised controlled trials of dietary interventions. This is particularly true given under real-world conditions, people seeking to lose weight select their own dietary and/or physical activity approach(es). Moreover, in reality, switching between weight loss strategies will occur even when a particular approach has been suggested by a health professional or within randomised trials.

Two promising and popular dietary approaches, despite relatively little research around their use in humans, include paleolithic diets and intermittent fasting. Paleolithic diets are based on evolutionary principles and include meat, fruit, vegetables and nuts/seeds while eschewing grains, dairy, and processed foods. Early small studies have provided encouraging results [54–56]. However, whether overweight adults can adhere to paleolithic-type diets in the longer-term requires study. Intermittent fasting is usually defined as normal food intake 3–5 days a week and dramatically reduced intake (down to 2 MJ from typical intake of 8–10 MJ) for 2–4 days. Anecdotally this is believed to be much easier than reducing energy intake by a smaller amount (usually 2 MJ/day) *every* day, which forms part of the current guidelines. While a wealth of animal data supports the effectiveness of intermittent fasting for weight control [57], research in humans is less certain [58]. However, the small amount of data available does show modest evidence of effectiveness for treatment of obesity and cardioprotection [59, 60].

Exercise produces more successful weight loss than dietary change alone [61, 62], seems particularly important for weight maintenance [63] and has many additional health benefits, over and above that relating to energy balance. Regular physical exercise is associated with an approximate halving in the risk of cardiovascular disease [64], can decrease the incidence of diabetes by up to 50 % as part of lifestyle counselling [65], and even decrease insulin resistance in those with established metabolic syndrome [66]. However, a major public health challenge is how best to encourage people to be physically active on a regular basis. Although evidence that regular physical exercise is beneficial is overwhelming [67], adherence remains a major issue [68], with lack of time often cited as a major barrier [69]. Thus there has been increasing interest in ascertaining the minimum amount of exercise required that might produce effective health benefits. An alternative to meeting moderate to vigorous physical activity (MVPA) guidelines may be the promotion of high intensity interval training (HIIT). In HIIT, brief periods of high-intensity exercise are interposed with recovery periods at a much lower intensity [70]. Although HIIT regimes typically include 10 minutes or so of intense exercise performed three times per week, as little as 3 minutes of intense exercise per week has been shown to produce cardiovascular and metabolic improvements [71, 72], although effects on body composition are less certain [73]. Although HIIT holds promise, virtually all research to date has been conducted in the laboratory and it is uncertain whether these findings will translate into community settings. If people cannot complete HIIT by themselves, its efficacy as a public health approach is very limited. Whether participants can adhere to a HIIT training regime long-term is also not currently known.

### Aims and objectives

The goal of our study is to determine whether allowing participants to choose from a selection of appropriate diet and exercise plans, within the context of a randomised controlled trial evaluating four different support strategies, enhances adherence and promotes greater weight loss and positive health outcomes. Giving people the choice of which diet and exercise regime is best incorporated into their particular lifestyle is expected to improve adherence, reflects real-world conditions, and is consistent with the lack of evidence for meaningful differences between diet modalities. Acknowledging this concept in conjunction with testing different support strategies offers a unique opportunity to determine how best to support individuals to make dietary and exercise changes under real-world conditions.

The primary aim of our study is to determine the effectiveness of different support strategies (control condition, brief support, daily self-weighing, app use, hunger training) on weight loss at 12 and 24 months. Secondary aims are to determine:

(i) the effect of different support strategies on body composition, dietary intake, exercise, inflammatory markers, blood lipids and lipoproteins, adiponectin, ghrelin, and psychosocial indices at 12 and 24 months
(ii) the degree of adherence to each of the support strategies over the 24 months
(iii) the degree of adherence to each of the diet (Mediterranean, intermittent fasting, modified Paleo) and exercise (current recommendations, HIIT) plans that are selected over the 24 months and the outcomes within these self-selected groups.

## Methods/Design

### Study design

**Fig. 1:**
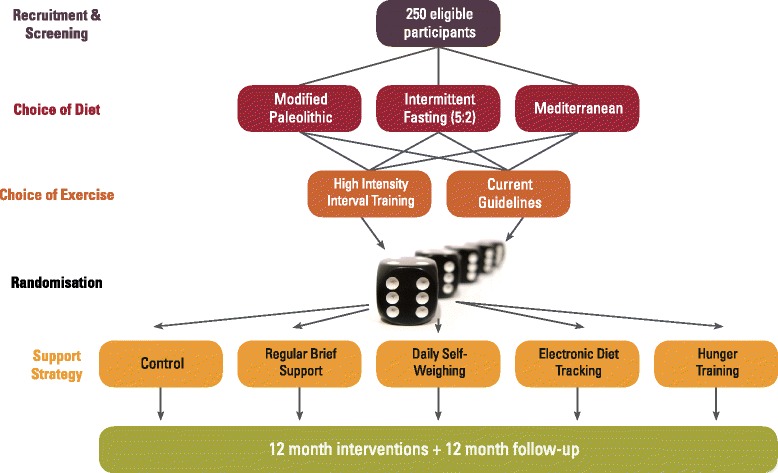
Overview of the study design including choice of diet/exercise plan and randomisation to support strategy

The Support strategies for Whole food diets, Intermittent Fasting and Training (SWIFT) study is a 5-arm randomised controlled trial testing the effectiveness of different support strategies for encouraging appropriate behaviour change for effective weight management. Participants will be randomised to one of the five groups for a 12-month intervention, with further follow-up at 24 months (Fig. 1) to determine whether any changes have been sustained. The primary analysis will be modified intention to treat (using all available data) and will focus on the outcomes resulting from the different support strategies (RCT analysis).

The trial has been approved by the University of Otago Human Ethics Committee (H14/024) and is registered with the Australian New Zealand Clinical Trials Registry ACTRN12615000010594. Written informed consent will be obtained from all participants before randomisation.

### Participants and recruitment

Recruitment will occur by advertisement (flyers, newspapers, email distribution lists) and word of mouth and interested people will be directed to complete an online screening questionnaire. They will be deemed eligible to attend a screening appointment if they indicate that they are at least 18 years of age, their self-reported body mass index (BMI) is greater than 27, they have internet access, they intend to remain in the local area for the duration of the 2-year intervention, and if female, they are not planning to become pregnant in next two years nor are currently breastfeeding, and have no history of cardiovascular or other serious medical conditions. Presence of symptoms suggesting undiagnosed heart disease and current medication use will be reviewed by medical staff. Further exclusions will occur for diabetes mellitus type 1 and 2, endocrine disorders, systemic inflammatory diseases and musculoskeletal disorders preventing exercise. People with stage one hypertension, dysglycaemia and mild controlled asthma will be potentially eligible.

### Screening appointment

Potentially eligible participants will attend a screening session following a 12-hour overnight fast. Duplicate measurements of height, weight, and systolic/diastolic blood pressure will be undertaken using standard techniques and venepuncture blood samples will be collected by a registered nurse. Participants will complete a comprehensive baseline questionnaire, and be instructed on how to complete a 3-day weighed diet record over the next week. Participants will also wear an Actigraph accelerometer for 7 days and nights to assess physical activity and sleep during the same time period. Further exclusion criteria will be applied at this point: measured BMI less than 27, fasting blood glucose greater than 7 mmol/L (if randomised to hunger training, fasting blood sugars consistently above 7 mmol/L would result in difficulties for the participant to adhere to hunger training guidelines), systolic BP greater than 160 mmHg or diastolic BP greater than 100 mmHg (because they require medical management for their hypertension). All participants will also undergo a dual-energy x-ray absorptiometry (DXA) scan at baseline (see outcome measures).

### Exercise safety screening

All participants will undergo medical screening by questionnaire to allow identification of those at higher risk of an adverse event during exercise. High-risk participants with known or likely occult heart disease will be excluded. Participants who choose high intensity regimes will be individually medically assessed and be stratified into categories of risk for cardiovascular events as per American College of Sports Medicine/American Heart Association (ACSM/AHA) guidelines [74]. Participants who are considered to be in a moderate-risk category who wish to participate in high-intensity exercise programmes will have a focussed medical examination, as per current ACSM guidelines.

### Choice of diet and exercise plan

If a participant is eligible and has completed all baseline assessments, they will be provided with information on each of the possible three dietary and two exercise approaches, and allowed to choose which might suit them best.

Their choice of dietary approaches will be:

1. *Mediterranean* – High amounts of fruit, vegetables and wholegrain cereals, moderate amounts of protein (particularly from fish), nuts (up to 30 g per day), olive oil and dairy foods, and limited amounts of processed and sugary foods. Calorie counting will not be required (unless randomised to MyFitnessPal), but energy intake should be reduced by appropriate levels through promotion of appropriate foods and serving sizes.
2. *Modified paleolithic* – Strict paleolithic diets remove all processed and cereal-based foods, legumes and dairy products which we believe is not sustainable for most overweight people long-term. Participants who choose this diet can decide to remove these foods; but we will also suggest they follow an 80:20 rule where up to one serving of dairy products, legumes and appropriate low glycaemic index, wholegrain carbohydrates are allowed each day. We believe this still fits the paleolithic philosophy while promoting greater long-term adherence through flexibility. Calorie counting will not be required (unless randomised to MyFitnessPal), but energy intake should be reduced by appropriate levels through promotion of appropriate foods and serving sizes.
3. *Intermittent fasting (5:2 plan)* – Participants choose two days per week (any days, not consecutive, can vary from week to week to fit lifestyle) where energy intake cannot exceed 2 (females) – 2.5 (males) MJ. In practice, this usually means a small breakfast (e.g. plain porridge), no or limited food during the day, and non-starchy vegetables only for the evening meal, although other variations are possible. Participants can eat *ad libitum* on the remaining days.

The choice of exercise approaches will be from:

1. *Current New Zealand guidelines* – recommend that participants engage in “at least 30 minutes of moderate intensity physical activity on most if not all days of the week. If possible, add some vigorous exercise for extra health benefit and fitness” (http://www.health.govt.nz/our-work/preventative-health-wellness/physical-activity). Standard printed resources available from the Ministry of Health will be used for counselling with this group. Typical recommended activities include walking briskly, exercise classes, and gardening but no mention is made of HIIT type activities (brief sessions of very high intensity exercise).
2. *Home-based high-intensity interval training (HIIT)* – Those choosing HIIT attend a private 1-hour training session which includes focused medical evaluation and HIIT training (cycle ergometer) using rating of perceived exertion in combination with heart rate monitoring. This typically includes 3 intervals of durations of up to 30 seconds, including two maximal sprint intervals. Fitness is likely to vary amongst participants at baseline, so clinical judgment is required to adapt the initial training of participants who have low baseline cardiorespiratory fitness or other significant issues influencing exercise tolerance. However, it is intended that all participants experience at least one observed interval that achieves 80-90 % of their estimated maximum heart rate which allows the participant to recognise the required intensity, and for further identification of any undisclosed cardiac symptoms. Participants then use heart rate monitors to record unsupervised HIIT sessions for another week, ensuring further confirmation that the required intensity is being achieved. Training and resources are then provided outlining how HIIT can be achieved at home. Four different protocols are provided including a “beginners” HIIT protocol (e.g. 10 second intervals at 90 % intensity repeated 3–5 times), and then three harder options. These include maximal (90 % maximum heart rate) and submaximal (80 %) options, involve a variety of interval lengths (e.g. 30 seconds to 4 minutes), and number of repeats (3–10 times) [70, 72, 75, 76]. In general, participants will be encouraged to gradually increase their HIIT from a beginners level to ultimately being able to complete three approximately 15-minute (allowing for warm-up and cool-down) sessions of HIIT each week following one of the submaximal or maximal options. A variety of possible exercises are suggested including sprinting, stair climbing, exercise equipment such as exercycles or rowing machines, and activities such as star jumps, burpees and the like, as long as it is “exercise that uses most of your body and is very hard to do within seconds”. Partictipants could also choose to do high intensity sports sessions that involved sprint intervals, or use commercial gym-based HIIT classes as acceptable alternatives.

An additional resource will be provided to all participants which focuses on evidence-based behavioural weight loss strategies known to be successful, including stimulus control, problem solving, stress reduction and dealing with negative thinking [77].

### Randomisation

Once participants have chosen their diet and exercise approach, randomisation will occur using sequentially numbered opaque sealed envelopes prepared by the statistician. The participants will be stratified by sex and random length blocks will be used to allocate the treatment. Participants will then be booked into their first intervention session.

### Intervention groups and sessions

1. *Control* – those randomised to the control condition will meet with research staff to discuss which diet and exercise options would suit them best. They will also receive the resource detailing the evidence-based behavioural weight loss strategies noted above. They will then be left to their own devices for the remainder of the study (except for attending all outcome assessments).
2. *Regular brief support* – Participants in this group will attend an appointment at the study clinic once a month to be weighed. During this time they will have the opportunity for a 5–10 minute conversation with research staff to assess progress, review and brainstorm solutions to problems if any exist, and encourage adherence. These sessions are modelled on our successful *HEAT* study and provide an opportunity for support and ongoing assistance with strategies [22].
3. *App* – Participants in the app group will attend an appointment to learn how to use MyFitnessPal to monitor their energy and macronutrient intakes. They will be provided with assistance in setting up their MyFitnessPal account to be compatible with their chosen diet, and will be shown how to use the app on their smartphone and/or computer. Participants will be asked to monitor their dietary intake every day for the first month, and for one week of every month for months 2–12.
4. *Daily self-weighing* – Individuals randomised to this group will receive instruction and support about weighing themselves every day (same time of day and degree of clothing). Participants will text their weight or enter it online using a web page connected to our secure database each day which will have a graphical display. Progress and adherence to entering/sending weight data will be checked every week and reminder texts sent where necessary. Every month, research staff will provide personalised progress feedback and support by email.
5. *Biochemical hunger training* – This group will follow a 4-week protocol that trains them to recognise “real” (biochemical) hunger by associating feelings of hunger with blood glucose levels following fingerprick testing with portable glucometers. Our protocol is based on that of Ciampolini et al. [45] but adapted slightly following piloting. In the original method, participants are only able to eat if blood glucose is less than 4.7 mmol/L [45]. Our pilot testing showed that use of an individualised blood glucose cut-off (average of fasting blood glucose over two days) rather than 4.7 mmol/L improved adherence to testing and reduced eating when blood glucose was not below the cut-off [78]. Before every desired eating occasion, participants will be instructed to note their intensity of hunger on a 100mm visual analogue scale and their measured blood glucose. If their blood glucose is higher than their personal cut-off, they are advised to engage in some other activity as a distraction and wait at least one hour. At this time, they assess their feelings of hunger again and repeat the measurement if they still want to eat, until their blood glucose is under their individualised cut-off. Over time, participants learn to relate physical feelings of hunger with their blood glucose and to eat only when physically hungry. Participants will be advised to follow this procedure for two weeks. In weeks 3 and 4, the blood glucose testing is optional, but all other recording (intensity and type of hunger, and resulting food intake) continues. Participants will be in regular contact with support staff, who will advise them how to proceed, answer any queries and provide encouragement. In months 2–12, participants will be advised to repeat the recording process (with or without fingerprick blood glucose testing) for one week of every month.

### Outcome assessments

Outcome assessments will occur at 0 (baseline), 6 (mid-point of intervention), 12 (end of intervention) and 24 (end of follow-up) months as shown in Table 1. Adherence measures are more frequent as outlined elsewhere.

**Table 1:** Timing of outcome assessments in the SWIFT study^*^

Outcome	Month			
	0^†^	6	12	24
Height	x			
Weight^§^	x	x	x	x
Bioimpedance	x	x	x	x
DXA scan	x		x	
Blood pressure	x	x	x	x
Blood samples	x		x	x
3-day diet record	x	x	x	x
Accelerometry	x	x	x	x
Aerobic fitness	x	x	x	x
Questionnaires	x	x	x	x
Demographics	x			
Personality	x			
Resilience	x			
Dieting and weight history	x			
Intuitive eating	x	x	x	x
Dutch eating behaviour questionnaire	x	x	x	x
Depression, anxiety, stress	x		x	x
Disordered eating	x		x	x
Self-monitoring	x	x	x	x
Self-efficacy	x	x	x	x
Satisfaction with diet and exercise		x	x	x

^*^Two visits are required at each time point in order to complete all measurements

^†^0 refers to baseline

^§^More frequent weights will be available for those in the regular brief support and daily self-weighing groups but these are for adherence measures rather than outcomes

#### Anthropometry and body composition

All measures (except DXA) will be obtained in duplicate by trained assessors blinded to support group allocation. If duplicate measures differ by more than 1 %, a third measurement is obtained and the median is used as the final value. Height will be measured by fixed stadiometer (Heightronic, QuickMedical, WA, USA) and weight by electronic scales (Tanita BC-418) with participants wearing light clothing and no shoes. Waist circumference will be measured at the narrowest point between the lower costal border and the top of the iliac crest by non-elastic tape. Body composition will be measured by segmental Bioelectric Impedance Analysis (BIA, Tanita BC-418) at each time point and by dual energy x-ray absorptiometry (Lunar Prodigy) at 0 and 12 months only. Measures of systolic and diastolic blood pressure will be obtained using an automated sphygmomanometer (Omron Model HEM-907).

#### Blood tests

Blood samples will be collected from participants by a registered nurse following a 12-hour overnight fast. High-sensitivity CRP will be measured using a CRP Unimate kit from Roche Diagnostics on a Cobas Mira Plus Analyzer (Roche), Interleukin-6 by using Quantikine ELISA Kits (R&D Systems) following the instructions provided by the manufacturer, adiponectin by radioimmunoassay (Linco Research, St Charles, MO, USA), ghrelin (active) by immunoassay (Human Gut Hormone Panel LINCOplex Kit, LINCO Research, St. Charles, MO, USA), and plasma total cholesterol (TC), HDL cholesterol (HDL-C), and TG concentrations by enzymatic methods using a Cobas Mira Plus Analyzer. LDL cholesterol (LDL-C) will be calculated using the Friedewald formula [79].

#### Diet, physical activity and fitness

Participants will complete a weighed 3-day diet record (one weekend day, two week days) with energy and nutrient intakes calculated using Kai-culator (University of Otago, 2011). Physical activity (counts per minute and intensity categories) and sleep duration and timing (minutes, bed time, wake time) will be measured using ActiGraph accelerometers (GT3X, ActiGraph, Pensacola, FL) worn around the waist over 7 days. Participants wear the accelerometers for the full 24-hour periods, which provides both sleep and activity data and lowers the chance of missing data from participants not remembering to reattach the accelerometer straight after waking. Aerobic fitness will be evaluated by estimating participants’ VO^2^max, using the YMCA submaximal cycle ergometer test [80].

#### Questionnaires

Demographic information (age, sex, education, ethnicity, employment, income, household structure) will be obtained at baseline using the relevant New Zealand census questions (http://www.stats.govt.nz/Census). Other questionnaires to be completed at baseline only include the Ten-item personality inventory [81] which gives broad scores for the ‘Big Five’ personality dimensions, the Brief Resilience scale [82] which assesses the ability to bounce back or recover from stress, and the Dieting and weight history questionnaire [83]. Questionnaires completed at baseline, and repeated at 6, 12 and 24 months (not all measures at all time points) will include the Intuitive Eating scale [84] which measures the tendency to follow hunger and satiety cues when eating, the Dutch Eating Behavior questionnaire [85] which evaluates dietary restraint and emotional and external eating, the Depression Anxiety Stress scale (DASS21) [86], a well-accepted short measure of depression, anxiety and stress, the Disordered Eating questionnaire EDE-Q [87] investigating restraint and concerns about eating, shape and weight, a self-monitoring questionnaire [39], assessing how often they weigh themselves and track their eating and physical activity, selected questions on perceived benefits, self-efficacy and enjoyment of physical activity, self-efficacy for health eating and behavioural skills used for weight management [88] and satisfaction with the dietary and exercise approaches chosen.

#### Adherence

Adherence to support strategies will be assessed as follows:

Brief support: attendance at monthly sessions.

App use: frequency, consistency, and comprehensiveness of food recording during first month (daily) and for one week every month for months 2–11 inclusive.

Daily-self weighing: by the number of daily weights recorded in the database.

Hunger training: analysis of the 4-week booklets in month 1 and the weekly recordings for months 2–12 the percentage of times participants measured their glucose before eating, and only ate if blood glucose was lower than the personal cut-off.

Adherence to the dietary regimes will be measured using the 3-day diet records. Those choosing to follow intermittent fasting will complete a 4-day diet record at 6, 12 and 24 months to allow collection of two fasting and two non-fasting days. Adherence to the exercise regime will be measured by the accelerometers. In addition, HIIT participants will wear a Polar RC3 GPS heart rate monitor during all home HIIT sessions for a one-week period at baseline, 3, 6, 9 and 12 months to evaluate intensity attained during the sessions.

### Statistical analyses and power calculations

Based on a standard deviation (SD) for baseline weight of 15 kg, and a correlation between repeat measures of r = 0.90 (obtained from our previous studies involving similar populations), our study has 90 % power using a two-sided 5 % level of significance to detect a clinically important difference in change in body weight of 4 kg between any pair of groups with 42 participants per group. While this may be viewed as a large difference, anything smaller does not really represent a difference of any importance between strategies. Thus we will recruit 250 participants in total across the five groups which allows for 15 % drop-out/unusable data. Fifty per group at baseline also provides 80 % power to detect differences of 5 cm in waist circumference (baseline SD 12, r = 0.80).

The primary analysis will follow modified intention-to-treat principles (using all available data) and will compare the outcomes resulting from the five different support strategies (*RCT analysis*). Linear mixed models will be used to model outcomes at 6, 12 and 24 months after adjusting for baseline values. Standard mixed model diagnostics will be performed. Although this analysis does not take diet and exercise choice into account because meta-analyses show there is little difference in outcomes from different treatments, further analysis adjusting for diet and exercise will be considered.

However, because participants do have choice over which diet and exercise plan they would like to follow, we are able to investigate the data in a number of ways. The baseline data will allow a *cross-sectional analysis* to assess the popularity of approaches among participants and then to examine what it is about people that lead them to choose to follow these different diet and exercise approaches (subject to sufficient numbers choosing each approach). Once the RCT analysis has been completed, we will be able to undertake a *cohort analysis* to determine whether adherence differs for each of the different diet and exercise approaches, subject to sufficient numbers making that particular choice, and how this differing adherence affects our study outcomes of interest.

All analyses will be performed using Stata 13.1 or a later version with all statistical tests performed at the two-sided 0.05 level.

## Discussion

Despite continued debate regarding which diet is best for weight loss, it is becoming increasingly apparent that a variety of possible diets, ranging in macronutrient content, are suitable healthy options [17]. A more pressing issue thus becomes determining how best to support people to follow one of these approaches [89]. Determining whether high-intensity exercise can be a viable public health approach to improving weight and health is also warranted, particularly given an intriguing recent finding that MVPA is more consistently associated with body weight than is diet quality [90]. The SWIFT trial aims to compare five (including a control group) different ways of helping people to follow one of several possible dietary and exercise combinations, a choice that we believe should enhance adherence and thus success with weight management. We believe our trial offers a pragmatic way of assessing whether simple support strategies, that require limited to no expert involvement, are viable ways for overweight adults to successfully manage their weight over a two-year period.
